# Supplementary material for: High-altitude is associated with better short-term survival in critically ill COVID-19 patients admitted to the ICU
Source: PLoS One. 2022 Mar 31;17(3):e0262423. doi: 10.1371/journal.pone.0262423 (PMC8970356; doi:10.1371/journal.pone.0262423)
Supplement: Attachment — Submitted filename: Point by Point Letter UCI Revision 2.docx [file pone.0262423.s002.docx]

Point by Point Letter

To:

Danielle R. Bruns, PhD

Academic Editor

PLOS ONE

RE: PONE-D-21-07862R1 “High-altitude is associated with better short-term survival in critically ill COVID-19 patients admitted to the ICU”

Dear Editor and reviewers, thank you very much for your effort, observing our manuscript for the second time. We have completed the revision and we have fulfilled your comments. All the changes are highlighted in red. We have also included a clean version and the rebuttal letter.

Additional Editor Comments:

Thank you for your revised manuscript. We appreciate the attention you took in addressing each reviewer's comments. I disclose that I participated as a reviewer for the initial evaluation of this manuscript. Upon second review, one reviewer still has a few concerns. While the manuscript is substantially improved, the major contribution of age in high- versus low-altitude communities is still inadequately addressed. Given the significant impact of age on COVID pathogenesis, we believe this is still a major concern in the revised version. The authors need to address this concern statistically (correct for the difference in age) or to at least discuss the importance of age in COVID-19.

Dear Editor, we have responded to your observations, and we believe that the further analysis improved our manuscript.

Secondly, some concerns remain with respect to the discussion of HIF-1, especially with the reference provided.

We have addressed this section and included a deeper analysis within the discussion section

Minor comments also remain regarding figure numbering.

We have reviewed the entire manuscript and corrected all the typos and mistakes while numbering tables and figures.

6. Review Comments to the Author

Reviewer #2: The authors have substantially improved the manuscript and most of my comments have been adequately addressed. However, 2 important concerns (+1 formal point) remain for me:

Thanks for your comments, we have improved our second revision

1. the difference in age distribution (much more individuals were in the highest age-categories (>56 years) in the low altitude group): although the problem is now mentioned in the limitations, this difference in distribution could explain the apparent altitude effect, if it were mostly the older people (>56) that died. I think it would be very important to correct for the age effect – at least the importance of age on mortality from COVID-19 has to be discussed. Currently the text reads, like age is a negligible factor in COVID-19 risk, which it clearly is not.

Thanks for your keen observation, we agreed with you, and we have corrected our findings after adjusting our results for age differences. The new analysis are highlighted in red and new surviving curves were elaborated

2. the argumentation about HIF-1 in high altitude dwellers. Unfortunately, the provided reference is behind a paywall (and most likely is not the ideal reference for this purpose), so it might be a confusion on my side: but the following statement needs clarification:

“High altitude patients present a chronic molecular conversion of the hypoxia-inducing factor type 1 to type 2 (HIF-1), which favors a greater tolerance to hypoxemia and decreases the acute tissue damage triggered by patients with severe acute respiratory conditions41”

I am not sure, what is meant with “conversion of HIF-1 type 1 to type 2”. Does this simply refer to increased stabilization of HIF-1alpha in high altitude residents? Does it refer to the interaction of HIF1- and HIF-2 pathways? Or to genetic adaptations in HIF-2alpha associated with its enhanced activity in some high altitude populations (such as in Tibetans)? If any of these effects are eluded to, the sentence needs to be more precise (a review that would provide adequate references is for example this one: Bigham & Lee. Human high-altitude adaptation: forward genetics meets the HIF pathway. Genes Dev. 2014). Otherwise, I think better explanation has to be provided for what is meant with “type 1 and type 2”.

Many thanks for your comments, we have deepened our discussion on this very subject and clarified the doubt raised by you.

3. In addition, please note, that all figures are still labeled Fig. 1.

Thanks for this observation, we have numbered them accordingly.
